# Performance of anthropometric indices in early detection of diabetes and prediabetes in the Brazilian population: a cross-sectional study, Brazil, 2013

**DOI:** 10.1590/S2237-96222026v35e20240606.en

**Published:** 2026-07-04

**Authors:** Luís Antônio Batista Tonaco, Mariana Santos Felisbino-Mendes, Alexandra Dias Moreira, Amanda Maria de Sousa Romeiro, Erika Aparecida Silveira, Maria José Silva Souza, Gustavo Velásquez-Meléndez

**Affiliations:** 1 Universidade Federal de Minas Gerais, Departamento Materno-infantil e Saúde Pública, Belo Horizonte, MG, Brazil Universidade Federal de Minas Gerais Departamento Materno-infantil e Saúde Pública Belo Horizonte MG Brazil; 2 Universidade Federal de Goiás, Faculdade de Medicina, Goiânia, GO, Brazil Universidade Federal de Goiás Faculdade de Medicina Goiânia GO Brazil

**Keywords:** Prediabetic State, Diabetes Mellitus, ROC Curve, Cross-Sectional Studies, Anthropometry., Estado Prediabético, Diabetes Mellitus, Curva ROC, Estudios Transversales, Antropometría.

## Abstract

**Objective:**

To assess the diagnostic performance of anthropometric adiposity metrics for diabetes and prediabetes detection in Brazilian adults.

**Methods:**

This was a cross-sectional study using data from national surveys, such as glycated hemoglobin laboratory test data. Diabetes was defined as glycated hemoglobin ≥6.5% or when hypoglycemic agents were being taken; prediabetes was defined as glycated hemoglobin ≥5.7% and <6.5%. Diabetes and prediabetes prevalence rates were estimated with 95% confidence intervals (95%CI). The diagnostic performance of anthropometric indices - body mass index (BMI), waist circumference (WC), waist-to-height ratio (WHR), and body shape index - were evaluated using the area under the ROC curve (AUC), sensitivity, specificity, positive and negative predictive value, and Youden's index, stratified by sex and age group.

**Results:**

8,435 individuals participated in the study. Diabetes prevalence was 8.6% (95%CI 7.8; 9.3), while prediabetes prevalence was 16.2% (95%CI 15.1; 17.3). In males <40 years old, the AUC for the anthropometric indices was close to 0.5; among males between 40 and 59 years old, it ranged from 0.66 to 0.71; in older males, the AUC for WC was 0.72. Among females <40 years old, the WHR AUC was 0.77; for those between 40 and 59 years old, it ranged from 0.64 to 0.69; and in older females, the WC AUC was 0.66. The AUCs were lower for prediabetes: in males, they ranged from 0.58 to 0.63; while in females they ranged from 0.60 to 0.65.

**Conclusion:**

WC was the index with the best performance for differentiating individuals with diabetes, while the ROC curve showed greater accuracy among adults <60 years old.

## Resumen

**Objetivo:**

Evaluar el rendimiento diagnóstico de los índices antropométricos de adiposidad para la detección de diabetes y prediabetes en adultos brasileños.

**Métodos:**

Estudio transversal con datos de encuestas nacionales, como análisis de laboratorio de hemoglobina glucosilada. La diabetes se definió como hemoglobina glucosilada ≥6,5% o cuando se utilizaron hipoglucemiantes; la prediabetes se definió como hemoglobina glucosilada ≥5,7% y <6,5%. Las prevalencias de diabetes y prediabetes se estimaron con intervalos de confianza del 95% (IC95%). El rendimiento diagnóstico de los índices antropométricos - índice de masa corporal (IMC), circunferencia de la cintura (CC), índice cintura-talla (ICT) e índice de forma corporal - se evaluó mediante el área bajo la curva ROC (AUC), la sensibilidad, la especificidad, el valor predictivo positivo y negativo, y el índice de Youden, estratificados por sexo y grupo de edad.

**Resultados:**

8.435 individuos participaron en el estudio. La prevalencia de diabetes fue del 8,6% (IC95%: 7,8; ​​9,3) y la de prediabetes del 16,2% (IC95%: 15,1; 17,3). En hombres <40 años, el AUC de los índices antropométricos fue cercano a 0,5; entre 40 y 59 años, osciló entre 0,66 y 0,71; en hombres mayores, el CC tuvo un AUC de 0,72. En mujeres <40 años, el AUC del ICT fue de 0,77; entre 40 y 59 años, osciló entre 0,64 y 0,69; y en mujeres mayores, el AUC del CC fue de 0,66. Para la prediabetes, las AUC fueron menores: en hombres, oscilaron entre 0,58 y 0,63; En mujeres, de 0,60 a 0,65.

**Conclusión:**

La CC fue el índice con mejor rendimiento para discriminar a las personas con diabetes, mientras que la curva ROC mostró mayor precisión en adultos menores de 60 años.

**Ethical aspects**

This research respected ethical principles, having obtained the following approval data: Research ethics committee: Comissão Nacional de Ética em Pesquisa. Opinion number: 328,159. Approval date: 26/6/2013. Certificate of submission for ethical appraisal: 10853812.7.0000.0008. Informed consent record: Obtained from all participants prior to data collection.

## Introduction 

Diabetes has a significant impact on the global burden of disease. In 2021, worldwide there were 537 million people with diabetes and projections indicate that there will be 780 million in 2045^1^. When uncontrolled, diabetes can increase the risk of cardiovascular disease, nephropathy, neuropathy, retinopathy and result in increased hospitalizations^2^.

Overweight is one of the main determinants of diabetes^3^. Accumulation of adipose tissue, above the normal level, contributes to the pathophysiological process of insulin resistance, responsible for initiating the causal chain of generalized metabolic outcomes, such as hypertension, hyperinsulinemia, diabetes and dyslipidemia, conditions collectively known as metabolic syndrome^4^. Thus, it is believed that anthropometric indices indicating excess accumulation of adipose tissue can be useful as diabetes screening and prevention indicators^5^^,^^6^.

Body mass index (BMI) is widely used as an estimate of nutritional status and is frequently employed in diagnosis of obesity, both in clinical and population contexts^7^. Its use has limitations, as it does not distinguish between the components of body mass and those of accumulated peripheral adipose tissue^8^^,^^9^.

Waist circumference (WC) allows for an indirect measurement of the amount of fat located in the abdomen and is used as one of the components of metabolic syndrome^10^. It can also incorporate height into its calculation, which generates the waist circumference-to-height ratio (WHR)^11^. A meta-analysis involving 300,000 participants from studies published between 1950 and 2008, identified that WHR was the best indicator among cardiometabolic risk factors^3^, associated with the same outcomes^3^.

Anthropometric metrics are easy to take, non-invasive and low cost^12^. Among other composite metrics, the body shape index (BSI) has been proposed, which incorporates WC, height and BMI^13^. This indicator has been shown to be a good index of body fat accumulation^14^.

There is no global consensus, nor even consensus in Brazil, on the cut-off points for anthropometric metrics that can identify adults at risk of metabolic disorders. Studies carried out with Brazilians, conducted with local or non-representative samples of the population^15^^,^^16^, and with other populations, have already demonstrated that these metrics are associated with metabolic diseases, such as diabetes^6^.

This study aimed to assess the diagnostic performance of different anthropometric metrics of adiposity for diabetes and prediabetes detection in Brazilian adults using data from the 2013 National Health Survey.

## Methods 

Design 

This is a cross-sectional study, with data from the 2013 National Health Survey. Laboratory test data were collected in 2014 and 2015, and the two databases were linked.

Setting

The 2013 National Health Survey was conducted by the Brazilian Institute of Geography and Statistics in collaboration with the Ministry of Health and the Oswaldo Cruz Foundation. It is a population-based survey, with a representative sample of the Brazilian population, which aims to provide national-level data on the health status and lifestyle habits of the population, as well as information related to health care, such as access to and use of health services, prevention actions, continuity of care and health care funding^17^.

Participants

All adults aged 18 years or older who answered the National Health Survey in 2013 and who had biological samples collected in 2014-2015 were included in the study.

Variables

The following were included as independent variables: sex (male; female), race/skin color (White; Black; Asian; Brazilian mixed-race; Indigenous), age group (in years: 18-30; 31-40; 41-50; 51-60; 61-104), schooling (from illiterate to complete elementary; incomplete and complete high school; incomplete and complete higher education), region of residence (North; Northeast; Southeast; South; Midwest), alcohol consumption (non-drinker; light/moderate; excessive), smoking (non-smoker; former smoker; smoker), leisure-time physical activity (yes; no), measured hypertension (yes; no), self-reported heart disease (yes; no), altered high-density lipoprotein (yes: ≤40 mg/dL in males and ≤50 mg/dL in females; no: >40 mg/dL in males and >50 mg/dL in females). Self-rated health (good and excellent; poor, very poor; fair), altered waist circumference (yes: ≥88 cm in females and ≥102 cm in males; no: <88 cm in females and <102 cm in males), altered waist-to-height ratio (yes: ≥0.5; no: <0.5) and body mass index (underweight: <18.5; healthy weight: ≥18.5 and <25; overweight and obesity: ≥25).

Data sources and measurement 

Regarding diabetes as the outcome, pregnant women, women who did not know whether or not they were pregnant, and individuals who had not undergone a glycated hemoglobin test were excluded. Regarding prediabetes at the outcome, individuals with diabetes (glycated hemoglobin ≥6.5% and those taking oral hypoglycemic agents and insulin) were not considered, as these conditions could overlap.

Participants were defined as diabetic when they had serum glycated hemoglobin levels ≥6.5% or were using medication to control diabetes^18^, as per the answers to the question: "In the last two weeks, because of diabetes, did you: Take oral medications to lower your blood sugar?/Use insulin?". Prediabetes was determined when individuals presented glycated hemoglobin levels ≥5.7 and <6.5%^18^.

For the purpose of anthropometric measurements, participants were instructed to be barefoot, without heavy clothing or accessories. Weight was measured using portable digital scales. Height was measured using a portable stadiometer. Waist circumference (WC) was measured with an anthropometric tape measure at the midpoint between the last rib and the iliac crest, without clothing around the waist. Interviewees stood with their arms flexed and crossed in front of their chests, feet apart and abdomen relaxed. Interviewees was asked to inhale and exhale completely until the measurement was taken. All measurements followed international recommendations, were performed three times, and average measurements were obtained^19^.

Based on anthropometric metrics of weight, height and WC, it was possible to build the following variables: body mass index (BMI) (weight/height²), waist-to-height ratio (WHR) (waist circumference in cm/height in cm), and body shape index (BSI). The latter was calculated using WC adjusted for height and BMI^13^. WC itself was also used. BMI was categorized as underweight (<18.5), healthy weight (≥18.5 and <25), and overweight and obesity (≥25). Altered WHR was dichotomized into no (<0.5) and yes (≥0.5). Altered WC was defined as follows: no (<88 cm for females and <102 cm for males men) and yes (≥88 cm for females and ≥102 cm for males).

The smoking variable (non-smoker; former smoker and smoker) was built from the following questions: "Do you currently smoke any tobacco product?" and "And in the past, did you smoke any tobacco product?".

The alcohol consumption variable (non-drinker; light/moderate; excessive) was consolidated from the following questions: "How many days a week do you usually drink alcoholic beverage?" and "In general, on the day you drink, how many doses of alcoholic beverage do you consume?"^20^.

Physical activity was defined as at least 150 minutes of moderate-intensity sports activities per week or 75 minutes of vigorous-intensity leisure activities per week. This variable was built from the following questions: "How many days a week do you usually exercise or play sports?", "In general, on the day you exercise or play sports, how many hours does this activity last?", and "In general, on the day you exercise or play sports, how many minutes does this activity last?".

The "heart disease" variable (yes; no) was built from the question: "Has a doctor ever diagnosed you with a heart disease such as a heart attack, angina, heart failure, or other heart disease?".

The altered high-density lipoprotein variable was built based on the following categories: no (>40 mg/dL for males and >50 mg/dL for females) and yes (≤40 mg/dL for males and ≤50 mg/dL for females)^21^.

Study size 

The sampling plan for the 2013 National Health Survey was developed using three-stage cluster sampling^17^. The primary sampling units were selected from the Brazilian Institute of Geography and Statistics master sample and correspond to census tracts^17^. The second stage consisted of selecting households in each primary sampling unit. The third stage corresponded to the random selection of the household resident^17^. A questionnaire was administered by means of face-to-face interviews, as well as anthropometric measurements and blood pressure level checks, among individuals aged ≥18 years and residing in urban or rural areas of Brazil^17^. From the total number of household interviews, a subsample of 25% was extracted for biological material and biochemical analysis.

Statistical analysis 

Descriptive analysis was performed based on sociodemographic and lifestyle variables, and anthropometric metrics. Diabetes and prediabetes prevalence rates and their respective 95% confidence intervals (95%CI) were estimated. Descriptive data analyses were performed using the Stata 14.0 survey module, which includes complex sample data structures for population estimates.

Diagnostic performance assessment was conducted using MedCalc Statistical Software version 16.4.3. For each anthropometric index, Receiver Operating Characteristics (ROC) curves were built, allowing estimation of cut-off points with the best diagnostic performance and the largest area under the curve. Other parameters were estimated to assess anthropometric index diagnosis in diabetes detection: sensitivity, specificity, positive and negative predictive values, and Youden's index. All analyses were stratified by sex (male; female) and age group (18 to 40; ≥40 to 60; ≥60 years).

## Results 

The final sample of individuals who had biological material collected and who met the study's inclusion criteria was 8,435. Diabetes prevalence was 8.6% (95%CI 7.8; 9.3) and was even higher in the population that self-identified as Indigenous (13.9%; 95%CI 6.8; 25.8), in those aged ≥60 years (20.9%; 95%CI 18.8; 23.3), with low education levels (11.7%; 95%CI 10.6; 12.9), former smokers (13.5%; 95%CI 11.5; 15.7), those who self-reported having received a medical diagnosis of heart disease (20.7%; 95%CI 16.2; 26.0) and those with low high-density lipoprotein cholesterol (11.3%; 95%CI 10.2; 12.5) (Table 1).

Prediabetes prevalence was 16.2% (95%CI 15.1; 7.3). The highest prediabetes prevalence rates were found in those aged ≥60 years (32.8%; 95%CI 29.9; 35.8), those with low education levels (20.7%; 95%CI 19.1; 22.3), former smokers (21.3%; 95%CI 18.7; 24.1), those who did not practice physical activities (16.8%; 95%CI 15.6; 18.1), those with hypertension (24.5%; 95%CI 32.9; 27.3) and those with low high-density lipoprotein cholesterol (19.6%; 95%CI 18.0; 21.3) (Table 1).

The diagnostic performance indicators of anthropometric indices for differentiating diabetes by age group and sex are presented in Table 2. The area under the curve for all anthropometric metrics was close to 0.5 in the male population aged <40 years. The area under the curve values ​​were close to 0.7 in the ≥40 to <60 year age group, with the exception of the body shape index (BSI), which was 0.6. Waist circumference (WC) stood out in terms of sensitivity, specificity and positive predictive value in the elderly population.

The areas under the curve were above 0.75 in females aged <40 years, except for the BSI (Table 2). The waist-to-height ratio (WHR) stood out with good balance between sensitivity and specificity (both 71%) and a higher positive predictive value (Figure 1; Table 3). The areas under the curve were greater than 0.64 in the ≥40 and <60 year age range, except for the BSI (0.55). The WHR showed a positive predictive value of 18% in this age range. The areas under the curve were greater than 0.63 in the elderly group, except for the BSI (0.55), with emphasis on the positive predictive values ​​of the BMI, WC and WHR (Table 2).

The diagnostic performance indicators of anthropometric metrics for prediabetes differentiation are presented in Table 3 and Figure 2. The area under the curve for all anthropometric metrics was close to 0.5 in the male population aged <40 years. The area under the curve values ​​were close to 0.62 in the ≥40 to <60 years age range, except for the BSI, which was 0.5. The area under the curve values ​​were also close to 0.6 in the elderly population, except for the BSI (0.52), with WC standing out in terms of positive predictive value.

The areas under the curve were above 0.6 in women in the <40 age group, except for the BSI (Table 3). The areas under the curve for all participants were above 0.62 in the ≥40 and <60 age groups, except for the BSI (0.54) (Table 3). The areas under the curve for all anthropometric metrics were close to 0.5 in the elderly group (Table 3).

**Table 1:** Diabetes and prediabetes prevalence and 95% confidence intervals in the adult population, according to sociodemographic and lifestyle characteristics and anthropometric metrics. Brazil, 2014-2015 (n=8,435)

Variables	Total			Diabetes (glycated hemoglobin ≥6.5% or taking medication)		Total			Prediabetes (glycated hemoglobin ≥5.7 and <6.5)	
	%	95%CI	%	95%CI	p-value	%	95%CI	%	95%CI	p-value
Total	100	-	8.6	(7.8-9.3)		100	-	16.2	(15.1-17.3)	
Sex					<0.001					0.023
Male	47.8	(46.4; 49.3)	7.0	(6.1; 8.1)		48.6	(47.1; 50.2)	14.9	(13.4; 16.6)	
Female	52.1	(50.7; 53.6)	10.0	(8.9; 11.1)		51.3	(49.8; 52.8)	17.4	(16.0; 18.9)	
Race/skin color					0.201					<0.001
White	47.8	(46.6; 49.3)	8.7	(7.6; 9.9)		47.8	(46.2; 49.3)	16.2	(14.6; 17.9)	
Black	9.3	(8.5; 10.2)	10.4	(7.9; 13.6)		9.1	(8.3; 10.1)	23.6	(19.4; 28.5)	
Asian	0.6	(0.4; 0.9)	4.7	(1.8; 11.6)		0.7	(0.5; 01.0)	17.7	(6.3; 40.7)	
Mixed race	41.9	(40.5; 43.3)	8.0	(7.1; 9.1)		42.1	(40.7; 43.6)	14.4	(13.1; 15.9)	
Indigenous	0.3	(0.2; 0.4)	13.9	(6.8; 25.8)		0.3	(0.2; 0.4)	29.8	(13.7; 53.1)	
Age group (years)					<0.001					<0.001
18-30	21.5	(20.2; 22.8)	1.7	(1.0; 2.9)		23.1	(21.7; 24.6)	6.0	(04.5; 07.9)	
31-40	21.7	(20.6; 23.0)	2.3	(1.5; 3.4)		23.2	(22.0; 24.6)	8.8	(07.1; 10.7)	
41-50	19.4	(18.3; 20.5)	6.0	(04.7; 07.7)		19.9	(18.8; 21.1)	14.0	(11.9; 16.5)	
51-60	16.5	(15.6; 17.6)	13.1	(11.1; 15.4)		15.7	(14.7; 16.8)	26.1	(23.0; 29.4)	
61-104	20.8	(19.7; 21.9)	20.9	(18.8; 23.3)		18.0	(16.9; 19.0)	32.8	(29.9; 35.8)	
Schooling					<0.001					<0.001
From illiterate to complete elementary	49.3	(47.8; 50.7)	11.7	(10.6; 12.9)		47.6	(46.1; 49.1)	20.7	(19.1; 22.3)	
Incomplete and complete high school	33.8	(32.4; 35.2)	5.9	(4.8; 07.2)		34.8	(33.3; 36.3)	12.2	(10.6; 14.1)	
Incomplete and complete higher education	16.9	(15.8; 18.1)	4.8	(3.6; 06.4)		17.6	(16.4; 18.9)	12.0	(9.8; 14.7)	
Region					0.018					0.015
North	6.9	(6.6; 07.3)	6.3	(5.4; 07.5)		7.1	(6.7; 07.5)	13.2	(11.7; 14.8)	
Northeast	26.3	(25.2; 27.3)	7.8	(6.9; 8.8)		25.5	(25.4; 27.6)	15.0	(13.7; 16.5)	
Southeast	44.3	(42.8; 45.8)	9.5	(8.2; 11.1)		43.8	(42.2; 45.4)	17.6	(15.6; 19.8)	
South	15.0	(14.1; 15.6)	7.6	(6.2; 9.4)		15.1	(14.2; 16.2)	14.7	(12.5; 17.2)	
Midwest	7.5	(7.0; 8.0)	9.5	(7.8; 11.5)		7.4	(6.9; 8.0)	18.2	(15.6; 21.1)	
Alcoholic beverage consumption					<0.001					0.098
Non-drinker	75.2	(73.9; 76.5)	9.8	(8.9; 10.7)		74.2	(72.8; 75.6)	16.9	(15.7; 18.2)	
Light/moderate	18.1	(17.0; 19.3)	4.7	(3.6; 6.3)		18.9	(17.7; 20.2)	15.0	(12.6; 17.9)	
Abusive	6.6	(5.9; 7.5)	5.4	(3.4; 8.5)		6.9	(6.1; 7.7)	12.1	(8.4; 17.1)	
Tobacco smoking					<0.001					0.001
Non-smoker	67.1	(65.8; 68.5)	7.6	(6.8; 8.5)		67.8	(66.4; 69.2)	14.7	(13.5; 16.0)	
Former smoker	18.0	(16.9; 19.0)	13.5	(11.5; 15.7)		17.0	(16.0; 18.1)	21.3	(18.7; 24.1)	
Smoker	14.9	(13.9; 16.0)	6.9	(5.3; 8.8)		15.1	(15.1; 16.3)	17.1	(14.3; 20.4)	
Physical activity					<0.001					0.037
Yes	21.9	(20.7; 23.1)	5.8	(4.6; 7.3)		22.5	(21.2; 23.9)	14.0	(11.9; 16.4)	
No	78.1	(76.8; 79.3)	9.3	(8.5; 10.2)		77.5	(76.1; 78.7)	16.8	(15.6; 18.1)	
Measured hypertension					<0.001					<0.001
Yes	23.9	(22.7; 25.1)	15.1	(13.2; 17.1)		22.2	(21.0; 23.5)	24.5	(32.9; 27.3)	
No	76.1	(74.9; 77.3)	6.5	(5.8; 7.3)		77.8	(76.4; 79.0)	13.9	(12.7; 15.0)	
Self-reported heart disease					<0.001					<0.001
Yes	4.4	(03.9; 4.9)	20.7	(16.2; 26.0)		3.8	(3.3; 4.7)	31.7	(25.6; 38.4)	
No	95.6	(95.0; 96.1)	8.0	(07.3; 8.8)		96.2	(95.6; 96.7)	15.6	(14.5; 16.7)	
Altered high-density lipoprotein					<0.001					<0.001
Yes	52.7	(51.3; 54.2)	11.3	(10.2; 12.5)		51.2	(49.6; 52.7)	19.6	(18.0; 21.3)	
No	47.3	(51.3; 54.2)	5.6	(4.7; 6.6)		48.8	(47.3; 50.4)	13.0	(11.6; 14.5)	
Self-rated health					<0.001					<0.001
Good and excellent	64.9	(63.6; 62.2)	4.7	(4.1; 5.5)		67.6	(62.2; 68.9)	14.0	(12.8; 15.4)	
Poor, very poor and fair	35.1	(33.8; 36.4)	15.6	(14.1; 17.3)		32.4	(31.1; 33.8)	20.8	(18.9; 22.7)	
Altered waist circumference					<0.001					<0.001
Yes	39.3	(38.0; 40.7)	15.9	(14.4; 17.5)		36.2	(34.8; 37.6)	24.3	(22.4; 26.4)	
No	60.6	(59.3; 62.0)	3.8	(3.2; 4.5)		63.8	(62.4; 65.2)	11.6	(10.4; 12.9)	
Altered waist-to-height ratio					<0.001					<0.001
Yes	73.7	(73.4; 75.0)	11.0	(10.1; 12.0)		71.7	(26.9; 29.7)	19.7	(18.9; 21.1)	
No	26.3	(25.0; 27.6)	1.6	(1.1; 2.5)		28.3	(26.9; 29.7)	7.3	(6.0; 8.9)	
Body mass index					<0.001					<0.001
Underweight	2.6	(2.2; 3.2)	2.0	(0.8; 5.2)		02.8	(2.3; 3.4)	10.2	(6.2; 16.3)	
Healthy weight	38.9	(37.5; 40.3)	4.2	(3.4; 5.1)		40.1	(39.3; 42.3)	11.0	(9.6; 12.5)	
Overweight and obesity	58.5	(57.0; 59.9)	11.8	(10.7; 13.0)		56.4	(54.9; 57.9)	20.2	(18.8; 21.9)	

**Table 2:** Performance indicators of anthropometric indices for differentiating diabetes in the adult population, according to age and sex. Brazil, 2014-2015 (n=8,435)

Anthropometric indicators				
Age groups	Body mass index	Waist circumference	Waist-to-height ratio	Body shape index
Male				
Age <40 years				
Sensitivity (%)	63.0	33.3	22.2	81.5
Specificity (%)	54.2	80.2	91.0	30.1
Cut-off points	>25.8 Kg/m^2^	>97.3 cm	>0.6	≤0.13
Area under the curve (95%CI)	0.53 (0.52; 0.58)	0.56 (0.53; 0.58)	0.56 (0.53; 0.58)	0.50 (0.47; 0.53)
p-value	0.357	0.277	0.298	0.96
Youden's index	0.17	0.13	0.13	0.11
Positive predictive value (%)	2.81	3.42	4.93	2.39
Negative predictive value (%)	98.58	98.28	98.23	98.72
Age ≥40-60 years				
Sensitivity (%)	52.7	50.9	78.2	73.6
Specificity (%)	77.4	80.3	53.8	42.0
Cut-off points	>29.48 Kg/m^2^	>103.3 cm	>0.559	>0.13
Area under the curve (95%CI)	0.69 (0.66; 0.71)	0.71 (0.68; 0.73)	0.71 (0.69; 0.74)	0.58 (0.56; 0.61)
p-value	<0.001	<0.001	<0.001	<0.001
Youden's index	0.30	0.31	0.31	0.15
Positive predictive value (%)	16.41	17.87	12.48	9.65
Negative predictive value (%)	96.10	95.10	96.69	94.97
Age ≥60 years				
Sensitivity (%)	70.8	75.9	57.7	73.7
Specificity (%)	59.6	60.4	75.8	46.9
Cut-off points	>26.31 Kg/m^2^	>98.2 cm	>0.62	>0.14
Area under the curve (95%CI)	0.67 (0.63; 0.70)	0.71 (0.68; 0.74)	0.69 (0.66; 0.72)	0.62 (0.59; 0.66)
p-value	<0.001	<0.001	<0.001	<0.001
Youden's index	0.30	0.36	0.33	0.20
Positive predictive value (%)	25.68	27.42	31.97	21.48
Negative predictive value (%)	91.19	92.70	90.08	90.04
Female				
Age ≤40 years				
Sensitivity (%)	71.1	92.1	71.1	18.4
Specificity (%)	70.2	51.5	70.6	93.1
Cut-off points	>27.56 Kg/m^2^	>83.4 cm	>0.57	>0.14
Area under the curve (95%CI)	0.74 (0.72; 0.76)	0.76 (0.74; 0.78)	0.77 (0.75; 0.79)	0.54 (0.52; 0.56)
p-value	<0.001	<0.001	<0.001	0.329
Youden's index	0.41	0.43	0.41	0.11
Positive predictive value (%)	4.86	3.91	4.93	5.41
Negative predictive value (%)	99.12	99.67	99.12	96.15
Age ≥40-60 years				
Sensitivity (%)	67.6	64.8	60.4	64.8
Specificity (%)	55.3	64.6	70.3	45.5
Cut-off points	>27.39 Kg/m^2^	>94.2 cm	>0.61	>0.12
Area under the curve (95%CI)	0.64 (0.62; 0.66)	0.68 (0.66; 0.70)	0.69 (0.67; 0.71)	0.55 (0.53; 0.57)
p-value	<0.001	<0.001	<0.001	0.024
Youden's index	0.22	0.29	0.30	0.10
Positive predictive value (%)	13.94	16.40	17.89	11.30
Negative predictive value (%)	94.09	94.48	94.30	92.35
Age ≥60 years				
Sensitivity (%)	40.0	64.9	57.40	47.2
Specificity (%)	78.8	59.8	66.6	64.4
Cut-off points	>30.64 Kg/m^2^	>96.0 cm	>0.63	>0.13
Area under the curve (95%CI)	0.62 (0.59; 0.65)	0.65 (0.63; 0.68)	0.650 (0.62; 0.67)	0.55 (0.52; 0.58)
p-value	<0.001	<0.001	<0.001	0.008
Youden's index	0.18	0.24	0.22	0.11
Positive predictive value (%)	35.26	31.78	33.15	27.67
Negative predictive value (%)	81.98	85.51	84.41	80.86

**Figure 1:**
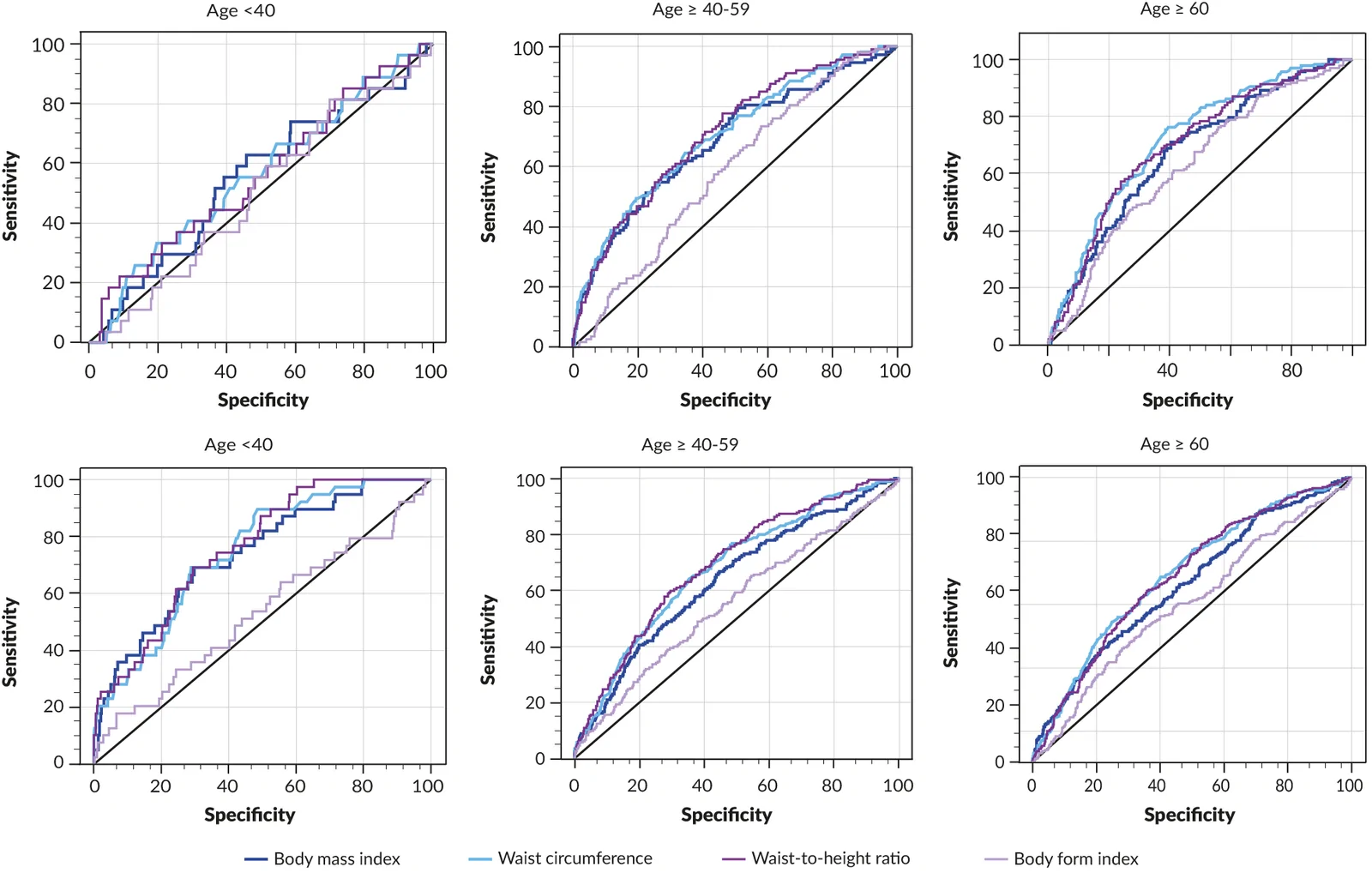
Area under the ROC curve of the anthropometric indices for diabetes diagnosis by age group in males <40 years old (A), in males ≥40 and ≥50 years old (B), in males ≥60 years old (C), in females <40 years old (D), in females ≥40 and ≥50 years old (E), and in females ≥60 years old (F). Brazil, 2014-2015 (n=8,435)

**Table 3:** Performance indicators of anthropometric indices for differentiating prediabetes in the adult population, according to age and sex. Brazil, 2014-2015 (n=7,675)

Anthropometric indicators				
Age groups	Body mass index	Waist circumference	Waist-to-height ratio	Body shape index
Male				
Age <40 years				
Sensitivity (%)	66.4	44.9	43.0	68.2
Specificity (%)	51.8	69.9	74.5	39.2
Cut-off points	>24.79 Kg/m^2^	>92.9 cm	>0.54	≤0.13
Area under the curve (95%CI)	0.59 (0.55; 0.61)	0.58 (0.54; 0.60)	0.58 (0.55; 0.61)	0.51 (0.48; 0.54)
p-value	<0.001	<0.001	0.007	0.7537
Youden's index	0.18	0.14	0.17	0.07
Positive predictive value (%)	11.13	11.95	13.30	9.26
Negative predictive value (%)	94.42	93.30	93.49	93.12
Age ≥40-60 years				
Sensitivity (%)	71.4	69.2	67.	75.00
Specificity (%)	45.5	50.3	52.1	29.3
Cut-off points	>25.50 Kg/m^2^	>93.0 cm	>0.55	>0.13
Area under the curve (95%CI)	0.61 (0.58; 0.63)	0.61 (0.58; 0.64)	0.62 (0.59; 0.65)	0.50 (0.48; 0.53)
p-value	<0.001	<0.001	<0.001	0.722
Youden's index	0.16	0.19	0.19	0.043
Positive predictive value (%)	21.31	22.39	22.68	18.01
Negative predictive value (%)	88.45	88.74	88.68	84.98
Age ≥60 years				
Sensitivity (%)	44.4	54.5	60.4	54.0
Specificity (%)	75.0	65.6	59.4	53.4
Cut-off points	>27.60 Kg/m^2^	>98.1 cm	>0.58	>0.14
Area under the curve (95%CI)	0.62 (0.58; 0.66)	0.62 (0.59; 0.66)	0.62 (0.59; 0.63)	0.52 (0.48; 0.55)
p-value	<0.001	<0.001	<0.001	0.425
Youden's index	0.19	0.20	0.19	0.07
Positive predictive value (%)	39.53	36.84	35.38	29.99
Negative predictive value (%)	78.55	79.65	80.29	75.98
Female				
Age <40 years				
Sensitivity (%)	43.1	61.8	58.5	26.83
Specificity (%)	75.1	54.8	57.	62.5
Cut-off points	>28.25 Kg/m^2^	>84.0 cm	>0.53	>0.12
Area under the curve (95%CI)	0.62 (0.59; 0.64)	0.61 (0.59; 0.63)	0.60 (0.57; 0.625)	0.50 (0.48; -0.53)
p-value	<0.001	<0.001	<0.001	0.774
Youden's index	0.18	0.16	0.16	0.10
Positive predictive value (%)	11.44	9.26	9.36	5.06
Negative predictive value (%)	94.64	95.05	94.90	91.95
Age ≥40-60 years				
Sensitivity	67.60	61.37	60.70	41.70
Specificity	54.80	62.48	62.60	65.50
Cut-off points	>26.87 Kg/m^2^	>92.2 cm	>0.59	>0.12
Area under the curve (95%CI)	0.63 (0.60; 0.65)	0.64 (0.62; 0.67)	0.65 (0.62; 0.67)	0.54 (0.51; 0.56)
p-value	<0.001	<0.001	<0.001	0.024
Youden's index	0.22	0.23	0.23	0.072
Positive predictive value (%)	25.83	27.58	27.43	21.96
Negative predictive value (%)	87.89	87.41	87.24	82.82
Age ≥60 years				
Sensitivity (%)	39.0	50.3	38.1	32.1
Specificity (%)	71.3	62.8	71.2	74.2
Cut-off points	>28.82 Kg/m^2^	>95.7 cm	>0.6422	>0.1351
Area under the curve (95%CI)	0.55 (0.51; 0.58)	0.55 (0.52; 0.58)	0.55 (0.52; 0.58)	0.51 (0.47; 0.54)
p-value	0.013	0.004	0.004	0.54
Youden's index	0.10	0.13	0.09	0.06
Positive predictive value (%)	41.86	41.74	41.21	39.73
Negative predictive value (%)	68.80	70.45	68.45	67.34

**Figure 2:**
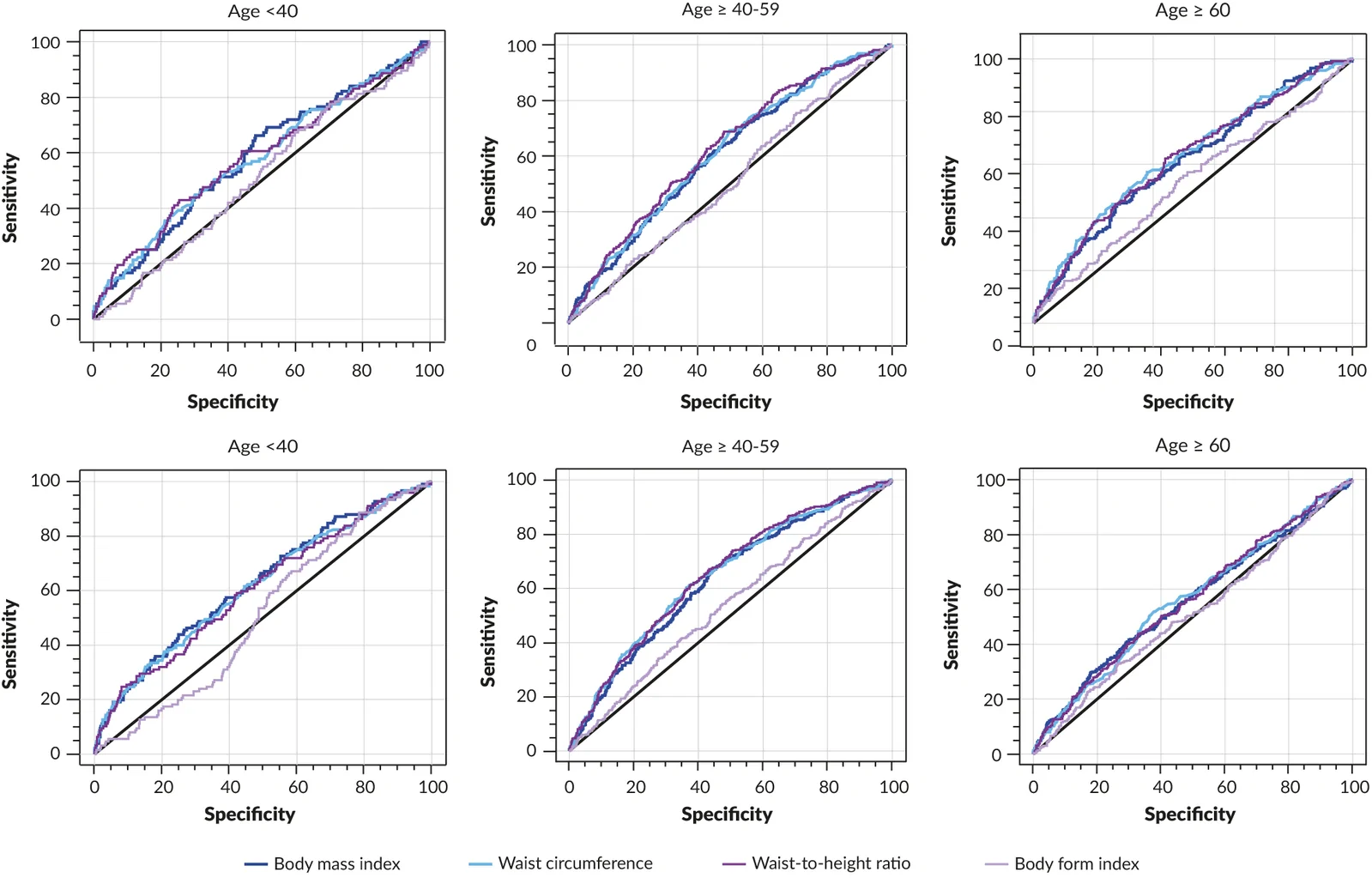
Area under the ROC curve of the anthropometric indices for prediabetes diagnosis by age group in males <40 years old (A), in males ≥40 and ≥50 years old (B), in males ≥60 years old (C), in females <40 years old (D), in females ≥40 and ≥50 years old (E), and in females ≥60 years old (F). Brazil, 2014-2015 (n=7,675)

## Discussion 

This study found that anthropometric indicators of central obesity, especially waist circumference (WC), showed better diagnostic performance for diabetes and prediabetes compared to overall obesity indices. Diagnostic capacity varied according to sex and age, with better performance in the group under 60 years old.

Standing out among the limitations of this study is its cross-sectional design, preventing the establishment of temporal relationships between anthropometric metrics and development of diabetes, thus limiting causal inferences. The absence of laboratory data in the 2019 National Health Survey, a similar representative survey, prevented the replication of the analysis with more recent data. Hip circumference was not measured, which could improve the accuracy of the models and reduce limitations. The possibility of information bias in sociodemographic variables should also be noted, although this would not have affected the analyses performed in this study.

While the World Health Organization recommends WC cut-off points ≥94 cm (males) and ≥80 cm (females) for increased risk, and ≥102 cm (males) and ≥88 cm (females) for substantially increased risk^22^, in this study, the best performance for differentiating diabetes was observed between 97 and 103 cm. These were rationally supported by the metabolic effects of obesity^18^^,^^23^ and were shown to be representative of the extent of visceral adiposity and increased risk of cardiovascular disease^24^. Reduction in WC measurements can be achieved through healthy behaviors^25^. This study demonstrated that the capacity to differentiate WC between sexes was similar, but with relatively different cut-off points, mainly in the <40 age group. This result can be explained by differences in body composition or distribution of body adipose tissue, body dimensions and other physiological events between the sexes, such as during menopause, a period in which changes occur in the distribution of abdominal adiposity^26^.

Stratified analysis by age group showed that, in older adults, WC remained the most sensitive indicator, which may be attributed to redistribution of body fat and loss of muscle mass that occurs with aging. The waist-to-height ratio (WHR) also showed good accuracy, but its operational complexity is greater than that of waist circumference on its own, especially in primary care contexts^3^.

The better performance of WC in diabetes screening was consistent with studies conducted in Latin American countries, such as Chile, in which WC showed similar performance in adult women^27^.

WC is an accessible, low-cost metric with good clinical acceptability^12^, but its incorporation is not yet systematic in health services, as evidenced in studies of medical records and prenatal cards. Measurement technique standardization and health professional training are essential for expanding its use as a cardiometabolic risk marker.

A systematic review of more than 120,000 individuals from 18 countries showed that WHR also performs well, especially in women^28^, although its applicability may be limited, mainly in the elderly population, which has potential height reduction due to musculoskeletal problems.

Analysis of 31 studies with both males and females confirmed that WHR association with diabetes and cardiovascular disease is similar to that of WC, sometimes even greater^28^. Inclusion of height in the WHR measurement is justified by the increased prediction of risk of cardiovascular disease, demonstrated in meta-analysis, due to short stature being directly associated with increased risk of development of coronary heart disease^29^.

The main contribution of this study was demonstrating that WC has relevant applicability for diabetes screening in the general population, based on representative national data and using glycated hemoglobin as the gold standard for diabetes diagnosis^18^. This reinforces its adoption as a risk marker in primary care protocols, with the potential to guide preventive actions even before diagnosis, especially in more vulnerable groups.

## Data Availability

The data used to produce this article are available in: Meléndez-Velásquez JG. Desempenho dos índices antropométricos na detecção precoce de Diabetes e Pré-diabetes na população brasileira. 2025 [cited 2025 Jul 05]. Available from: https://dx.doi.org/10.17605/OSF.IO/GYTEC.
