# Supplementary material for: Performance of anthropometric indices in early detection of diabetes and prediabetes in the Brazilian population: a cross-sectional study, Brazil, 2013
Source: Epidemiol Serv Saude. 2026 Jul 4;35:e20240606. doi: 10.1590/S2237-96222026v35e20240606.en (PMC13686399; doi:10.1590/S2237-96222026v35e20240606.en)
Supplement: Translation

# Translation: Desempenho dos índices antropométricos na detecção precoce de diabetes e pré-diabetes na população brasileira: estudo transversal, Brasil, 2013

**DOI:** 10.1590/S2237-96222026v35e20240606.pt

**Authors:** Luís Antônio Batista Tonaco, Mariana Santos Felisbino-Mendes, Alexandra Dias Moreira, Amanda Maria de Sousa Romeiro, Erika Aparecida Silveira, Maria José Silva Souza, Gustavo Velásquez-Meléndez

**Affiliations:** 1 Universidade Federal de Minas Gerais, Departamento Materno-infantil e Saúde Pública, Belo Horizonte, MG, Brasil Universidade Federal de Minas Gerais Departamento Materno-infantil e Saúde Pública Belo Horizonte MG Brasil; 2 Universidade Federal de Goiás, Faculdade de Medicina, Goiânia, GO, Brasil Universidade Federal de Goiás Faculdade de Medicina Goiânia GO Brasil

**Keywords:** Estado Pré-Diabético, Diabetes Mellitus, Curva ROC, Estudos Transversais, Antropometria.

## Abstract

**Objetivo:**

Avaliar o desempenho diagnóstico de medidas antropométricas de adiposidade para detecção de diabetes e pré-diabetes em adultos brasileiros.

**Métodos:**

Estudo transversal com dados de inquéritos nacionais, como exames laboratoriais de hemoglobina glicada. Considerou-se diabetes com hemoglobina glicada ≥6,5% ou quando havia uso de hipoglicemiantes; pré-diabetes com hemoglobina glicada ≥5,7% e <6,5%. As prevalências de diabetes e pré-diabetes foram estimadas com os intervalos de confiança de 95% (IC95%). O desempenho diagnóstico dos índices antropométricos - índice de massa corporal (IMC), circunferência da cintura (CC), razão circunferência/altura (RCE) e índice de forma corporal - foram avaliados por meio da área sob a curva ROC (AUC), a sensibilidade, a especificidade, o valor preditivo positivo e negativo e o índice de Youden, estratificados por sexo e faixa etária**.**

**Resultados:**

8.435 indivíduos participaram do estudo. A prevalência de diabetes foi 8,6% (IC95% 7,8; 9,3), e a de pré-diabetes 16,2% (IC95% 15,1; 17,3). Em homens <40 anos, a AUC dos índices antropométricos foi próxima de 0,5; entre 40 e 59 anos, variou de 0,66 a 0,71; em idosos, a CC apresentou AUC de 0,72. Em mulheres <40 anos, a AUC da RCE foi 0,77; entre 40 e 59 anos, variou de 0,64 a 0,69; e em idosas, a AUC da CC foi 0,66. Para pré-diabetes, as AUCs foram menores: em homens, variaram de 0,58 a 0,63; em mulheres, de 0,60 a 0,65.

**Conclusão:**

A CC foi o índice de melhor desempenho para discriminar indivíduos com diabetes, enquanto a RCE mostrou maior acurácia entre adultos <60 anos.

Aspectos éticos

Esta pesquisa respeitou os princípios éticos, obtendo os seguintes dados de aprovação: Comitê de ética em pesquisa: Comissão Nacional de Ética em Pesquisa. Número do parecer: 328.159. Data de aprovação: 26/6/2013. Certificado de apresentação de apreciação ética: 10853812.7.0000.0008. Registro do consentimento livre e esclarecido: Obtido de todos os participantes antes da coleta.

## Introdução 

O diabetes exerce um impacto significativo na carga global de doenças. Em 2021, existiam 537 milhões de diabéticos no mundo e as projeções apontam para 780 milhões em 2045^1^. Quando não controlado, o diabetes pode elevar o risco de doenças cardiovasculares, nefropatia, neuropatia, retinopatia e resultar em um aumento nas hospitalizações^2^.

O excesso de peso é um dos principais determinantes do diabetes^3^. O acúmulo de tecido adiposo, acima do nível normal, contribui para o processo fisiopatológico da resistência à insulina, responsável por iniciar a cadeia causal para desfechos metabólicos generalizados, como hipertensão, hiperinsulinemia, diabetes e dislipidemia, condições conjuntas denominadas de síndrome metabólica^4^. Dessa forma, acredita-se que os índices antropométricos indicadores de excesso de acúmulo de tecido adiposo podem ser úteis como indicadores de rastreamento e de prevenção do diabetes^5^^,^^6^.

A medida do índice de massa corporal (IMC) é amplamente utilizada como uma estimativa do estado nutricional e frequentemente é empregada no diagnóstico da obesidade, tanto em contextos clínicos quanto populacionais^7^. A sua utilização possui limitações, por não distinguir os componentes da massa corporal e os do acúmulo da localização periférica do tecido adiposo^8^^,^^9^.

A circunferência da cintura (CC) permite aferir indiretamente a dimensão da gordura localizada em nível abdominal e é utilizada como um dos componentes da síndrome metabólica^10^. Ela também pode incorporar no seu cálculo a altura, o que gera o índice de circunferência da cintura/altura (RCE)^11^. Em uma meta-análise com 300 mil participantes de estudos publicados entre 1950 e 2008, a RCE foi identificada como o melhor indicador entre os fatores de risco cardiometabólicos^3^, associada aos mesmos desfechos^3^. 

As medidas antropométricas são de fácil aferição, não invasivas e de baixo custo^12^. Entre outras medidas compostas, tem sido proposto o índice de forma corporal (IFC), que incorpora a CC para altura e o IMC^13^. Esse indicador foi apontado como um bom índice de acúmulo de gordura corporal^14^.

Não há consenso global, nem entre a população brasileira, sobre os pontos de corte para medidas antropométricas que possam identificar adultos em risco de alterações metabólicas. Estudos realizados com brasileiros, conduzidos com amostras locais ou não representativas da população^15^^,^^16^, e com outras populações já demonstraram que essas medidas estão associadas a doenças metabólicas, como o diabetes^6^. 

Este estudo visou avaliar o desempenho diagnóstico de diferentes medidas antropométricas de adiposidade para detecção de diabetes e de pré-diabetes em adultos brasileiros a partir de dados da Pesquisa Nacional de Saúde de 2013.

## Métodos 

Desenho do estudo

Trata-se de estudo transversal, com dados provenientes da Pesquisa Nacional de Saúde (PNS) de 2013. Os dados dos exames laboratoriais foram coletados em 2014 e em 2015, e as duas bases de dados foram relacionadas. 

Contexto

A PNS foi realizada pelo Instituto Brasileiro de Geografia e Estatística em colaboração com o Ministério da Saúde e a Fundação Oswaldo Cruz. Trata-se de um inquérito populacional, com amostra representativa da população brasileira, que tem como objetivo fornecer dados em nível nacional sobre a situação de saúde e os hábitos de vida da população, além de informações relacionadas à atenção à saúde, como o acesso e a utilização dos serviços, as ações de prevenção, a continuidade dos cuidados e o financiamento da assistência de saúde^17^.

Participantes

Foram incluídos no estudo todos os indivíduos adultos com ≥18 anos que responderam a PNS em 2013 e que tiveram amostras de material biológico coletada em 2014-2015.

Variáveis

Foram incluídas como variáveis independentes: sexo (masculino; feminino), raça/cor da pele (branca; preta; amarela; parda; indígena), faixa etária (em anos: 18 a 30; 31 a 40; 41 a 50; 51 a 60; 61 a 104), escolaridade (sem instrução até fundamental completo; médio incompleto e médio completo; superior incompleto e superior completo), região de residência (Norte; Nordeste; Sudeste; Sul; Centro-Oeste), consumo de bebida alcoólica (não bebe; leve/moderado; abusivo), tabagismo (não fuma; ex-fumante; fumante), prática de atividade física no lazer (sim; não), hipertensão arterial aferida (sim; não), doença do coração autorreferida (sim; não), lipoproteína de alta densidade alterada (sim: ≤40 mg/dL em homens e ≤50 mg/dL em mulheres; não: >40 mg/dL em homens e >50 mg/dL em mulheres), autopercepção de saúde (boa e ótima; ruim, muito ruim; regular), circunferência da cintura alterada (sim: ≥88 cm nas mulheres e ≥102 cm em homens; não: <88 cm nas mulheres e <102 cm em homens), razão circunferência/altura alterada (sim: ≥0,5; não: <0,5) e índice de massa corporal (baixo peso: <18,5; eutrófico: ≥18,5 e <25; sobrepeso e obesidade: ≥25). 

Fontes de dados e mensuração

Para o desfecho de diabetes foram excluídas as mulheres gestantes, as que não sabiam se estavam grávidas e os indivíduos que não tinham realizado exame de hemoglobina glicada. Para o desfecho de pré-diabetes, não foram considerados os indivíduos com diabetes (hemoglobina glicada ≥6,5% e pessoas que faziam uso de hipoglicemiante oral e de insulina), pois essas condições poderiam se sobrepor.

Participantes com dosagem sérica de hemoglobina glicada ≥6,5% ou em uso de medicação para controle do diabetes, implicados pela pergunta: "Nas duas últimas semanas, por causa do diabetes, o(a) sr(a): Tomou medicamentos orais para baixar o açúcar?/Usou insulina?", foram definidos como diabéticos^18^. O pré-diabetes foi determinado quando os indivíduos apresentaram hemoglobina glicada ≥5,7 e <6,5%^18^.

Para aferição da antropometria, os participantes foram orientados a ficar descalços, sem roupas pesadas e sem acessórios. O peso foi aferido com a utilização de balança portátil e digital. A estatura foi aferida por meio de estadiômetro portátil. A circunferência da cintura (CC) foi aferida com o auxílio de uma fita antropométrica no ponto médio entre a última costela e a crista ilíaca, sem roupas na região da cintura. O entrevistado permaneceu com braços flexionados e cruzados à frente do tórax, pés afastados e abdômen relaxado. Para aferição, solicitou-se que o entrevistado inspirasse e soltasse completamente o ar dos pulmões até que fosse realizada a leitura da medida. Todas as medidas seguiram recomendações internacionais, foram realizadas três vezes e tiveram obtenção da suas médias^19^.

Com base nas medidas antropométricas de peso, de altura e de CC, foi possível construir as seguintes variáveis: índice de massa corporal (IMC) (peso/altura^2^), razão circunferência/altura (RCE) (circunferência da cintura em cm/estatura em cm) e índice de forma corporal (IFC). Este último foi calculado pela CC ajustada pela altura e pelo IMC^13^. Utilizou-se também a própria CC. O IMC foi categorizado em baixo peso (<18,5), eutrófico (≥18,5 e <25) e excesso de peso e obesidade (≥25). A RCE alterada foi dicotomizada em não (<0,5) e em sim (≥0,5). A CC alterada foi constituída da seguinte forma: não (<88 cm nas mulheres e <102 cm em homens) e sim (≥88 cm nas mulheres e ≥102 cm em homens).

A variável tabagismo (não fuma; ex-fumante e fumante) foi construída a partir das seguintes perguntas: "Atualmente, o(a) sr(a) fuma algum produto do tabaco?" e "E no passado, o(a) sr(a) fumou algum produto do tabaco?".

A variável consumo de álcool (não bebe; leve/moderado; abusivo) foi consolidada a partir das seguintes perguntas: "Quantos dias por semana o(a) sr(a) costuma tomar alguma bebida alcoólica?" e "Em geral, no dia que o(a) sr(a) bebe, quantas doses de bebida alcoólica o(a) sr(a) consome?"^20^.

A prática de atividade física foi considerada com pelo menos 150 minutos de atividades esportivas por semana de forma moderada ou com 75 minutos por semana de atividades de lazer de forma vigorosa. Essa variável foi construída a partir das seguintes questões: "Quantos dias por semana o(a) sr(a) costuma praticar exercício físico ou esporte?", "Em geral, no dia que o(a) sr(a) pratica exercício ou esporte, quantas horas dura esta atividade?" e "Em geral, no dia que o(a) sr(a) pratica exercício ou esporte, quantos minutos dura esta atividade?".

A variável doença do coração (sim; não) foi construída a partir da pergunta: "Algum médico já lhe deu o diagnóstico de uma doença do coração tais como infarto, angina, insuficiência cardíaca ou outra?".

A lipoproteína de alta densidade alterada foi construída com o seguimento das seguintes categorias: não (>40 mg/dL em homens e >50 mg/dL em mulheres) e sim (≤40 mg/dL em homens e ≤50 mg/dL em mulheres)^21^.

Tamanho do estudo

O plano amostral da PNS 2013 foi desenvolvido por amostragem conglomerada em três estágios^17^. As unidades primárias de amostragem foram selecionadas a partir da amostra mestra do Instituto Brasileiro de Geografia e Estatística e correspondem aos setores censitários^17^. O segundo estágio foi composto a partir da seleção dos domicílios em cada unidade primária de amostragem. Já o terceiro estágio correspondeu à seleção aleatória do residente^17^. Foi aplicado questionário por entrevista face-a-face, bem como aferição de medidas antropométricas e dos níveis pressóricos, em indivíduos com ≥18 anos e residentes da área urbana ou rural do Brasil^17^. Do total de entrevistas domiciliares, foi extraída uma subamostra para material biológico e análise bioquímica de 25%.

Análise estatística

A análise descritiva foi realizada segundo variáveis sociodemográficas, estilo de vida e medidas antropométricas . Foram estimadas as prevalências de diabetes e de pré-diabetes e os seus respectivos intervalos de confiança de 95% (IC95%). As análises descritivas dos dados foram realizadas com a utilização do software Stata 14.0 no módulo survey, que contempla estrutura amostral complexa dos dados para estimativas populacionais.

A avaliação do desempenho e do diagnóstico for realizada com auxílio do programa estatístico MedCalc Statistical Sofware version 16.4.3. Para cada índice antropométrico, foram construídas as curvas Receiver Operator Characteristics, o que permitiu estimar os pontos de corte com melhor desempenho diagnóstico e maior área sob a curva. Outros parâmetros foram estimados para avaliação diagnóstica dos índices antropométricos na detecção de diabetes: sensibilidade, especificidade, valor preditivo positivo e negativo e índice de Youden. Todas as análises foram estratificadas por sexo (masculino; feminino) e faixa etária (18 a 40; ≥40 a 60; ≥60 anos).

## Resultados 

A amostra final de indivíduos que tiveram material biológico coletado e que cumpriram o critério de inclusão do estudo foi de 8.435. A prevalência de diabetes foi 8,6% (IC95% 7,8; 9,3) e foi ainda mais alta na população que autodeclarou raça/cor da pele indígena (13,9%; IC95% 6,8; 25,8), na com idade ≥60 anos (20,9%; IC95% 18,8; 23,3), com baixa escolaridade (11,7%; IC95% 10,6; 12,9), na população de ex-tabagistas (13,5%; IC95% 11,5; 15,7), na que autodeclarou ter recebido diagnóstico médico de doença do coração (20,7%; IC95% 16,2; 26,0) e na população com baixo colesterol de lipoproteína de alta densidade (11,3%; IC95% 10,2; 12,5) (Tabela 1).

A prevalência de pré-diabetes foi 16,2% (IC95% 15,1; 7,3). As maiores prevalências de pré-diabetes foram observadas na população com idade ≥60 anos (32,8%; IC95% 29,9; 35,8), na com baixa escolaridade (20,7%; IC95% 19,1; 22,3), na população de ex- fumantes (21,3%; IC95% 18,7; 24,1), de indivíduos que não praticavam atividade física (16,8%; IC95% 15,6; 18,1), na população com hipertensão (24,5%; IC95% 32,9; 27,3) e na com baixo colesterol de lipoproteína de alta densidade (19,6%; IC95% 18,0; 21,3) (Tabela 1).

Os indicadores de desempenho diagnóstico dos índices antropométricos para discriminar diabetes por faixa etária e por sexo são apresentados na Tabela 2. A área sob a curva de todas as medidas antropométricas foi próxima de 0,5 na população masculina com idade <40 anos. Os valores das áreas sob a curva ficaram próximos de 0,7 na faixa etária ≥40 a <60 anos, com exceção do índice de forma corporal (IFC), que foi 0,6. O destaque foi para a circunferência da cintura (CC) em termos de sensibilidade, de especificidade e de valor preditivo positivo na população de idosos.

As áreas sob a curva ficaram acima de 0,75 nas mulheres com idade <40 anos, exceto para o IFC (Tabela 2). Destaca-se a razão circunferência/altura (RCE) com bom equilíbrio entre sensibilidade e especificidade (71% ambas) e valor mais alto de valor preditivo positivo (Figura 1; Tabela 3). As áreas sob a curva foram maiores que 0,64 na faixa etária ≥40 e <60 anos, exceto para o IFC (0,55). A RCE apresentou valor preditivo positivo de 18% nessa faixa etária. As áreas sob a curva foram maiores de 0,63 no grupo de idosas, exceto para o IFC (0,55), com destaque para os valores preditivos positivos do IMC, da CC e da RCE (Tabela 2).

Os indicadores de desempenho diagnóstico das medidas antropométricas para discriminação de pré-diabetes são apresentados na Tabela 3 e na Figura 2. A área sob a curva de todas as medidas antropométricas foi próxima de 0,5 na população masculina com idade <40 anos. Os valores das áreas sob a curva ficaram próximos de 0,62 na faixa etária ≥40 a <60 anos, com exceção do IFC, que foi 0,5. Os valores das áreas sob a curva também ficaram próximos de 0,6 na população de idosos, exceto para o IFC (0,52), com destaque para a CC em termos de valor preditivo positivo.

As áreas sob a curva ficaram acima de 0,6 nas mulheres na faixa etária <40 anos, exceto no caso do IFC (Tabela 3). As áreas sob a curva de todos ficaram acima de 0,62 na faixa etária ≥40 e <60 anos, com exceção do IFC (0,54) (Tabela 3). As áreas sob a curva de todas as medidas antropométricas foram próximas de 0,5 no grupo de idosas (Tabela 3).

**Tabela 1:** Prevalência e intervalos de confiança de 95% (IC95%) de diabetes e de pré-diabetes, segundo características sociodemográficas, estilo de vida e medidas antropométricas da população adulta. Brasil, 2014-2015( n=8.435)

Variáveis	Total			Diabetes (hemoglobina glicada ≥6,5% ou em uso de medicamentos)		Total			Pré-diabetes (hemoglobina glicada ≥5,7 e <6,5)	
	%	IC95%	%	IC95%	p-valor	%	IC95%	%	IC95%	p-valor
Total	100	-	8,6	(7,8-9,3)		100	-	16,2	(15,1-17,3)	
Sexo					<0,001					0,023
Masculino	47,8	(46,4; 49,3)	7,0	(6,1; 8,1)		48,6	(47,1; 50,2)	14,9	(13,4; 16,6)	
Feminino	52,1	(50,7; 53,6)	10,0	(8,9; 11,1)		51,3	(49,8; 52,8)	17,4	(16,0; 18,9)	
Raça/cor da pele					0,201					<0,001
Brancaz	47,8	(46,6; 49,3)	8,7	(7,6; 9,9)		47,8	(46,2; 49,3)	16,2	(14,6; 17,9)	
Preta	9,3	(8,5; 10,2)	10,4	(7,9; 13,6)		9,1	(8,3; 10,1)	23,6	(19,4; 28,5)	
Amarela	0,6	(0,4; 0,9)	4,7	(1,8; 11,6)		0,7	(0,5; 01,0)	17,7	(6,3; 40,7)	
Parda	41,9	(40,5; 43,3)	8,0	(7,1; 9,1)		42,1	(40,7; 43,6)	14,4	(13,1; 15,9)	
Indígena	0,3	(0,2; 0,4)	13,9	(6,8; 25,8)		0,3	(0,2; 0,4)	29,8	(13,7; 53,1)	
Faixa etária (anos)					<0,001					<0,001
18-30	21,5	(20,2; 22,8)	1,7	(1,0; 2,9)		23,1	(21,7; 24,6)	6,0	(04,5; 07,9)	
31-40	21,7	(20,6; 23,0)	2,3	(1,5; 3,4)		23,2	(22,0; 24,6)	8,8	(07,1; 10,7)	
41-50	19,4	(18,3; 20,5)	6,0	(04,7; 07,7)		19,9	(18,8; 21,1)	14,0	(11,9; 16,5)	
51-60	16,5	(15,6; 17,6)	13,1	(11,1; 15,4)		15,7	(14,7; 16,8)	26,1	(23,0; 29,4)	
61-104	20,8	(19,7; 21,9)	20,9	(18,8; 23,3)		18,0	(16,9; 19,0)	32,8	(29,9; 35,8)	
Nível de escolaridade					<0,001					<0,001
Sem instrução até fundamental completo	49,3	(47,8; 50,7)	11,7	(10,6; 12,9)		47,6	(46,1; 49,1)	20,7	(19,1; 22,3)	
Médio incompleto e médio completo	33,8	(32,4; 35,2)	5,9	(4,8; 07,2)		34,8	(33,3; 36;3)	12,2	(10,6; 14,1)	
Superior incompleto e superior completo	16,9	(15,8; 18,1)	4,8	(3,6; 06,4)		17,6	(16,4; 18,9)	12,0	(9,8; 14,7)	
Região					0,018					0,015
Norte	6,9	(6,6; 07,3)	6,3	(5,4; 07,5)		7,1	(6,7; 07,5)	13,2	(11,7; 14,8)	
Nordeste	26,3	(25,2; 27,3)	7,8	(6,9; 8,8)		25,5	(25,4; 27,6)	15,0	(13,7; 16,5)	
Sudeste	44,3	(42,8; 45,8)	9,5	(8,2; 11,1)		43,8	(42,2; 45,4)	17,6	(15,6; 19,8)	
Sul	15,0	(14,1; 15,6)	7,6	(6,2; 9,4)		15,1	(14,2; 16,2)	14,7	(12,5; 17,2)	
Centro-Oeste	7,5	(7,0; 8,0)	9,5	(7,8; 11,5)		7,4	(6,9; 8,0)	18,2	(15,6; 21,1)	
Consumo de bebida alcóolica					<0,001					0,098
Não bebe	75,2	(73,9; 76,5)	9,8	(8,9; 10,7)		74,2	(72,8; 75,6)	16,9	(15,7; 18,2)	
Leve/moderado	18,1	(17,0; 19,3)	4,7	(3,6; 6,3)		18,9	(17,7; 20,2)	15,0	(12,6; 17,9)	
Abusivo	6,6	(5,9; 7,5)	5,4	(3,4; 8,5)		6,9	(6,1; 7,7)	12,1	(8,4; 17,1)	
Tabagismo					<0,001					0,001
Não fuma	67,1	(65,8; 68,5)	7,6	(6,8; 8,5)		67,8	(66,4; 69,2)	14,7	(13,5; 16,0)	
Ex-fumante	18,0	(16,9; 19,0)	13,5	(11,5; 15,7)		17,0	(16,0; 18,1)	21,3	(18,7; 24,1)	
Fumante	14,9	(13,9; 16,0)	6,9	(5,3; 8,8)		15,1	(15,1; 16,3)	17,1	(14,3; 20,4)	
Pratica atividade física					<0,001					0,037
Sim	21,9	(20,7; 23,1)	5,8	(4,6; 7,3)		22,5	(21,2; 23,9)	14,0	(11,9; 16,4)	
Não	78,1	(76,8; 79,3)	9,3	(8,5; 10,2)		77,5	(76,1; 78,7)	16,8	(15,6; 18,1)	
Hipertensão arterial aferida					<0,001					<0,001
Sim	23,9	(22,7; 25,1)	15,1	(13,2; 17,1)		22,2	(21,0; 23,5)	24,5	(32,9; 27,3)	
Não	76,1	(74,9; 77,3)	6,5	(5,8; 7,3)		77,8	(76,4; 79,0)	13,9	(12,7; 15,0)	
Doença do coração autorreferida					<0,001					<0,001
Sim	4,4	(03,9; 4,9)	20,7	(16,2; 26,0)		3,8	(3,3; 4,7)	31,7	(25,6; 38,4)	
Não	95,6	(95,0; 96,1)	8,0	(07,3; 8,8)		96,2	(95,6; 96,7)	15,6	(14,5; 16,7)	
Lipoproteína de alta densidade alterada					<0,001					<0,001
Sim	52,7	(51,3; 54,2)	11,3	(10,2; 12,5)		51,2	(49,6; 52,7)	19,6	(18,0; 21,3)	
Não	47,3	(51,3; 54,2)	5,6	(4,7; 6,6)		48,8	(47,3; 50,4)	13,0	(11,6; 14,5)	
Autopercepção de saúde					<0,001					<0,001
Boa e ótima	64,9	(63,6; 62,2)	4,7	(4,1; 5,5)		67,6	(62,2; 68,9)	14,0	(12,8; 15,4)	
Ruim, muito ruim e regular	35,1	(33,8; 36,4)	15,6	(14,1; 17,3)		32,4	(31,1; 33,8)	20,8	(18,9; 22,7)	
Circunferência da cintura alterada					<0,001					<0,001
Sim	39,3	(38,0; 40,7)	15,9	(14,4; 17,5)		36,2	(34,8; 37,6)	24,3	(22,4; 26,4)	
Não	60,6	(59,3; 62,0)	3,8	(3,2; 4,5)		63,8	(62,4; 65,2)	11,6	(10,4; 12,9)	
Razão circunferência/altura alterada					<0,001					<0,001
Sim	73,7	(73,4; 75,0)	11,0	(10,1; 12,0)		71,7	(26,9; 29,7)	19,7	(18,9; 21,1)	
Não	26,3	(25,0; 27,6)	1,6	(1,1; 2,5)		28,3	(26,9; 29,7)	7,3	(6,0; 8,9)	
Índice de massa corporal					<0,001					<0,001
Baixo peso	2,6	(2,2; 3,2)	2,0	(0,8; 5,2)		02,8	(2,3; 3,4)	10,2	(6,2; 16,3)	
Eutrófico	38,9	(37,5; 40,3)	4,2	(3,4; 5,1)		40,1	(39,3; 42,3)	11,0	(9,6; 12,5)	
Sobrepeso e obesidade	58,5	(57,0; 59,9)	11,8	(10,7; 13,0)		56,4	(54,9; 57,9)	20,2	(18,8; 21,9)	

**Tabela 2:** Indicadores de desempenho dos índices antropométricos para discriminar diabetes por idade e por sexo na população adulta. Brasil, 2014-2015 (n=8.435)

Grupos etários	Índice de massa corporal	Circunferência da cintura	Razão circunferência/altura	Índice de forma corporal
Masculino				
Idade <40 anos				
Sensibilidade (%)	63,0	33,3	22,2	81,5
Especificidade (%)	54,2	80,2	91,0	30,1
Pontos de corte	>25,8 Kg/m^2^	>97,3 cm	>0,6	≤0,13
Área sob a curva (IC95%)	0,53 (0,52; 0,58)	0,56 (0,53; 0,58)	0,56 (0,53; 0,58)	0,50 (0,47; 0,53)
p-valor	0,357	0,277	0,298	0,96
Índice de Youden	0,17	0,13	0,13	0,11
Valor preditivo positivo (%)	2,81	3,42	4,93	2,39
Valor preditivo negativo (%)	98,58	98,28	98,23	98,72
Idade ≥40-60 anos				
Sensibilidade (%)	52,7	50,9	78,2	73,6
Especificidade (%)	77,4	80,3	53,8	42,0
Pontos de corte	>29,48 Kg/m^2^	>103,3 cm	>0,559	>0,13
Área sob a curva (IC95%)	0,69 (0,66; 0,71)	0,71 (0,68; 0,73)	0,71 (0,69; 0,74)	0,58 (0,56; 0,61)
p-valor	<0,001	<0,001	<0,001	<0,001
Índice de Youden	0,30	0,31	0,31	0,15
Valor preditivo positivo (%)	16,41	17,87	12,48	9,65
Valor preditivo negativo (%)	96,10	95,10	96,69	94,97
Idade ≥60 anos				
Sensibilidade (%)	70,8	75,9	57,7	73,7
Especificidade (%)	59,6	60,4	75,8	46,9
Pontos de corte	>26,31 Kg/m^2^	>98,2 cm	>0,62	>0,14
Área sob a curva (IC95%)	0,67 (0,63; 0,70)	0,71 (0,68; 0,74)	0,69 (0,66; 0,72)	0,62 (0,59; 0,66)
p-valor	<0,001	<0,001	<0,001	<0,001
Índice de Youden	0,30	0,36	0,33	0,20
Valor preditivo positivo (%)	25,68	27,42	31,97	21,48
Valor preditivo negativo (%)	91,19	92,70	90,08	90,04
Feminino				
Idade ≤40 anos				
Sensibilidade (%)	71,1	92,1	71,1	18,4
Especificidade (%)	70,2	51,5	70,6	93,1
Pontos de corte	>27,56 Kg/m^2^	>83,4 cm	>0,57	>0,14
Área sob a curva (IC95%)	0,74 (0,72; 0,76)	0,76 (0,74; 0,78)	0,77 (0,75; 0,79)	0,54 (0,52; 0,56)
p-valor	<0,001	<0,001	<0,001	0,329
Índice de Youden	0,41	0,43	0,41	0,11
Valor preditivo positivo (%)	4,86	3,91	4,93	5,41
Valor preditivo negativo (%)	99,12	99,67	99,12	96,15
Idade ≥40-60 anos				
Sensibilidade (%)	67,6	64,8	60,4	64,8
Especificidade (%)	55,3	64,6	70,3	45,5
Pontos de corte	>27,39 Kg/m^2^	>94,2 cm	>0,61	>0,12
Área sob a curva (IC95%)	0,64 (0,62; 0,66)	0,68 (0,66; 0,70)	0,69 (0,67; 0,71)	0,55 (0,53; 0,57)
p-valor	<0,001	<0,001	<0,001	0,024
Índice de Youden	0,22	0,29	0,30	0,10
Valor preditivo positivo (%)	13,94	16,40	17,89	11,30
Valor preditivo negativo (%)	94,09	94,48	94,30	92,35
Idade ≥60 anos				
Sensibilidade (%)	40,0	64,9	57,40	47,2
Especificidade (%)	78,8	59,8	66,6	64,4
Pontos de corte	>30,64 Kg/m^2^	>96,0 cm	>0,63	>0,13
Área sob a curva (IC95%)	0,62 (0,59; 0,65)	0,65 (0,63; 0,68)	0,650 (0,62; 0,67)	0,55 (0,52; 0,58)
p-valor	<0,001	<0,001	<0,001	0,008
Índice de Youden	0,18	0,24	0,22	0,11
Valor preditivo positivo (%)	35,26	31,78	33,15	27,67
Valor preditivo negativo (%)	81,98	85,51	84,41	80,86

**Figura 1:** Área sob a curva ROC dos índices antropométricos para diagnóstico de diabetes por faixa etária em homens de <40 anos (A), em homens de ≥40 e de 50 anos (B), em homens de ≥60 anos (C), em mulheres de <40 anos (D), em mulheres de ≥40 e de 50 anos (E) e em mulheres de ≥60 anos (F). Brasil, 2014-2015 (n=8.435)

**Tabela 3:** Indicadores de desempenho dos índices antropométricos para discriminar pré-diabetes por idade e por sexo na população adulta. Brasil, 2014-2015 (n=7.675)

Indicadores antropométricos				
Grupos etários	Índice de massa corporal	Circunferência da cintura	Razão circunferência/altura	Índice de forma corporal
Masculino				
Idade <40 anos				
Sensibilidade (%)	66,4	44,9	43,0	68,2
Especificidade (%)	51,8	69,9	74,5	39,2
Pontos de corte	>24,79 Kg/m^2^	>92,9 cm	>0,54	≤0,13
Área sob a curva (IC95%)	0,59 (0,55; 0,61)	0,58 (0,54; 0,60)	0,58 (0,55; 0,61)	0,51 (0,48; 0,54)
p-valor	<0,001	<0,001	0,007	0,7537
Índice de Youden	0,18	0,14	0,17	0,07
Valor preditivo positivo (%)	11,13	11,95	13,30	9,26
Valor preditivo negativo (%)	94,42	93,30	93,49	93,12
Idade ≥40-60 anos				
Sensibilidade (%)	71,4	69,2	67,	75,00
Especificidade (%)	45,5	50,3	52,1	29,3
Pontos de corte	>25,50 Kg/m^2^	>93,0 cm	>0,55	>0,13
Área sob a curva (IC95%)	0,61 (0,58; 0,63)	0,61 (0,58; 0,64)	0,62 (0,59; 0,65)	0,50 (0,48; 0,53)
p-valor	<0,001	<0,001	<0,001	0,722
Índice de Youden	0,16	0,19	0,19	0,043
Valor preditivo positivo (%)	21,31	22,39	22,68	18,01
Valor preditivo negativo (%)	88,45	88,74	88,68	84,98
Idade ≥60 anos				
Sensibilidade (%)	44,4	54,5	60,4	54,0
Especificidade (%)	75,0	65,6	59,4	53,4
Pontos de corte	>27,60 Kg/m^2^	>98,1 cm	>0,58	>0,14
Área sob a curva (IC95%)	0,62 (0,58; 0,66)	0,62 (0,59; 0,66)	0,62 (0,59; 0,63)	0,52 (0,48; 0,55)
p-valor	<0,001	<0,001	<0,001	0,425
Índice de Youden	0,19	0,20	0,19	0,07
Valor preditivo positivo (%)	39,53	36,84	35,38	29,99
Valor preditivo negativo (%)	78,55	79,65	80,29	75,98
Feminino				
Idade <40 anos				
Sensibilidade (%)	43,1	61,8	58,5	26,83
Especificidade (%)	75,1	54,8	57,	62,5
Pontos de corte	>28,25 Kg/m^2^	>84,0 cm	>0,53	>0,12
Área sob a curva (IC95%)	0,62 (0,59; 0,64)	0,61 (0,59; 0,63)	0,60 (0,57; 0,625)	0,50 (0,48; -0,53)
p-valor	<0,001	<0,001	<0,001	0,774
Índice de Youden	0,18	0,16	0,16	0,10
Valor preditivo positivo (%)	11,44	9,26	9,36	5,06
Valor preditivo negativo (%)	94,64	95,05	94,90	91,95
Idade ≥40-60 anos				
Sensibilidade	67,60	61,37	60,70	41,70
Especificidade	54,80	62,48	62,60	65,50
Pontos de corte	>26,87 Kg/m^2^	>92,2 cm	>0,59	>0,12
Área sob a curva (IC95%)	0,63 (0,60; 0,65)	0,64 (0,62; 0,67)	0,65 (0,62; 0,67)	0,54 (0,51; 0,56)
p-valor	<0,001	<0,001	<0,001	0,024
Índice de Youden	0,22	0,23	0,23	0,072
Valor preditivo positivo (%)	25,83	27,58	27,43	21,96
Valor preditivo negativo (%)	87,89	87,41	87,24	82,82
Idade ≥60 anos				
Sensibilidade (%)	39,0	50,3	38,1	32,1
Especificidade (%)	71,3	62,8	71,2	74,2
Pontos de corte	>28,82 Kg/m^2^	>95,7 cm	>0,6422	>0,1351
Área sob a curva (IC95%)	0,55 (0,51; 0,58)	0,55 (0,52; 0,58)	0,55 (0,52; 0,58)	0,51 (0,47; 0,54)
p-valor	0,013	0,004	0,004	0,54
Índice de Youden	0,10	0,13	0,09	0,06
Valor preditivo positivo (%)	41,86	41,74	41,21	39,73
Valor preditivo negativo (%)	68,80	70,45	68,45	67,34

**Figura 2:** Área sob a curva ROC dos índices antropométricos para diagnóstico de pré-diabetes por faixa etária em homens de <40 anos (A), em homens de ≥40 e de 50 anos (B), em homens de ≥60 anos (C), em mulheres de <40 anos (D), em mulheres de ≥40 e de 50 anos (E), em mulheres de ≥60 anos (F). Brasil, 2014-2015 (n=7.675)

## Discussão 

Neste estudo foi observado que indicadores antropométricos de obesidade centralizada, especialmente a CC, apresentaram melhor desempenho diagnóstico para diabetes e para pré-diabetes em comparação com os índices de obesidade global. A capacidade diagnóstica variou segundo sexo e idade, com melhores desempenhos no grupo de <60 anos. 

Dentre as limitações deste estudo, destaca-se o delineamento transversal, que impede o estabelecimento de relações temporais entre os valores das medidas antropométricas e o desenvolvimento de diabetes, o que limita inferências causais. A ausência de dados laboratoriais na Pesquisa Nacional de Saúde (PNS) de 2019, pesquisa representativa similar, impediu a replicação da análise com dados mais recentes. A medida da circunferência do quadril não foi aferida, o que poderia aprimorar a acurácia dos modelos e diminuir limitações. Ressalta-se também a possibilidade de viés de informação nas variáveis sociodemográficas, o qual, pelo menos, não teria efeito nas análises realizadas neste estudo. 

Enquanto a OMS recomenda pontos de corte de CC ≥94 cm (homens) e ≥80cm (mulheres) para risco aumentado, e ≥102 cm (homens) e ≥88cm (mulheres) para risco substancialmente aumentado^22^, neste estudo, o melhor desempenho para discriminar diabetes foi observado entre 97 e 103 cm. Eles foram racionalmente apoiados pelos efeitos metabólicos da obesidade^18^^,^^23^ e se mostraram representativos da dimensão da adiposidade visceral e do aumento de risco para doenças cardiovasculares^24^. A redução nas medidas da CC pode ser alcançada com comportamentos saudáveis^25^.Foi demostrado neste estudo que a capacidade de discriminação da CC entre os sexos foi similar, mas com pontos de corte relativamente diferentes, principalmente na faixa etária <40 anos. Esse resultado pode ser explicado pelas diferenças na composição corporal ou na distribuição do tecido adiposo corporal, pelas dimensões corporais e por outros eventos fisiológicos entre os sexos, como no climatério, período em que ocorrem alterações na distribuição da adiposidade abdominal^26^. 

A análise estratificada por faixa etária mostrou que, em idosos, a CC continuou sendo o indicador mais sensível, o que pode ser atribuído à redistribuição da gordura corporal e à perda de massa muscular que ocorre com o envelhecimento. A razão circunferência/altura (RCE) também apresentou boa acurácia, mas sua complexidade operacional é maior do que a da circunferência isolada, principalmente em contextos de atenção primária^3^.

O melhor desempenho da CC no rastreamento de diabetes foi coerente com estudos realizados em países da América Latina, como o Chile, nos quais a CC apresentou desempenho semelhante em mulheres adultas^27^. 

A CC é uma medida acessível, de baixo custo e com boa aceitabilidade do ponto de vista clínico^12^, mas sua incorporação ainda não é sistemática em serviços de saúde, como evidenciado em estudos de prontuários e em cartões de pré-natal. A padronização da técnica de aferição e o treinamento de profissionais são essenciais para ampliar sua utilização como marcador de risco cardiometabólico.

Uma revisão sistemática com mais 120 mil indivíduos de 18 países apontou que a RCE também possui bom desempenho, sobretudo em mulheres^28^, ainda que sua aplicabilidade possa estar limitada principalmente na população de idosos, que apresenta potencial diminuição da altura devido a problemas osteomusculares. 

Na inclusão de homens e mulheres participantes de 31 estudos incluídos, confirmou-se que a RCE apresenta associação semelhante à CC, algumas vezes maior com o diabetes e com doenças cardiovasculares^28^. A inclusão da altura na medida da RCE se explica pelo aumento da previsão de risco da patologia, demonstrada em meta-análise, em decorrência da baixa altura estar diretamente associada à elevação do risco para o desenvolvimento de doenças cardiovasculares^29^.

A principal contribuição deste estudo foi demonstrar que a CC tem aplicabilidade relevante para o rastreamento de diabetes na população geral, com base em dados nacionais representativos e com uso de hemoglobina glicada como padrão ouro de diagnóstico da diabetes^18^. Isso reforça sua adoção como marcador de risco em protocolos de atenção primária, com potencial para orientar ações preventivas antes mesmo do diagnóstico, especialmente em grupos de maior vulnerabilidade.

## Data Availability

Os dados do artigo estão disponíveis em: Meléndez-Velásquez JG. Desempenho dos índices antropométricos na detecção precoce de Diabetes e Pré-diabetes na população brasileira. 2025 [cited 2025 Jul 05]. Available from: https://dx.doi.org/10.17605/OSF.IO/GYTEC.
